# Population genetic structure of the major malaria vector *Anopheles funestus s.s.* and allied species in southern Africa

**DOI:** 10.1186/1756-3305-5-283

**Published:** 2012-12-06

**Authors:** Kwang Shik Choi, Lizette L Koekemoer, Maureen Coetzee

**Affiliations:** 1 Malaria Entomology Research Unit, School of Pathology, Faculty of Health Sciences, University of the Witwatersrand, Johannesburg, South Africa; 2 Vector Control Reference Laboratory, Centre for Opportunistic, Tropical and Hospital Infections, National Institute for Communicable Diseases, National Health Laboratory Service, Johannesburg, South Africa

**Keywords:** Anopheles funestus, Clade, ND5, COI, Phylogeny

## Abstract

**Background:**

*Anopheles funestus s.s.*, one of the major malaria vectors in sub-Saharan Africa, belongs to a group of eleven African species that are morphologically similar at the adult stage, most of which do not transmit malaria. The population structure of *An. funestus* based on mitochondrial DNA data led to the description of two cryptic subdivisions, clade I widespread throughout Africa and clade II known only from Mozambique and Madagascar. In this study, we investigated five common members of the *Anopheles funestus* group in southern Africa in order to determine relationships within and between species.

**Methods:**

A total of 155 specimens of *An. funestus*, *An. parensis*, *An. vaneedeni*, *An. funestus*-like and *An. rivulorum* from South Africa, Mozambique and Malawi were used for the study. The population genetic structure was assessed within and between populations using mitochondrial DNA.

**Results:**

The phylogenetic trees revealed three main lineages: 1) *An. rivulorum*; 2) *An. funestus*-like clade I and *An. parensis* clade II; and 3) *An. funestus* clades I and II, *An. funestus*-like clade II, *An. parensis* clade I and *An. vaneedeni* clades I and II. Within *An. funestus*, 32 specimens from Mozambique consisted of 40.6% clade I and 59.4% clade II while all 21 individuals from Malawi were clade I. In the analysis of mitochondrial DNA sequences, there were 37 polymorphic sites and 9 fixed different nucleotides for *ND5* and 21 polymorphic sites and 6 fixed different nucleotides for *COI* between the two *An. funestus* clades. The results for *COI* supported the *ND5* analysis.

**Conclusion:**

This is the first report comparing *An. funestus* group species including *An. funestus* clades I and II and the new species *An. funestus*-like. *Anopheles funestus* clade I is separated from the rest of the members of the *An. funestus* subgroup and *An. funestus*-like is distinctly distributed from the other species in this study. However, there were two clades for *An. funestus*-like, *An. parensis* and *An. vaneedeni*. Further investigations are needed to determine what these results mean in terms of the specific status of the clades within each taxon and whether this has any epidemiological implications for malaria transmission.

## Background

Malaria due to *Plasmodium falciparum* is a major cause of morbidity and mortality in children and pregnant women. The World Health Organization estimates that there were 216 million malaria cases in 2010, with 655,000 deaths [1]. Malaria also poses a risk to travelers and immigrants, with imported cases increasing in non-endemic areas [2]. The treatment and control of malaria has become more difficult with the spread of drug-resistant strains of parasites [3] and insecticide-resistant mosquito vectors [4-6].

Depending on the vectorial capacity and competence of local mosquitoes, transmission intensity of human malaria varies across Africa. Only a limited number of *Anopheles* species are able to transmit *Plasmodium* malaria to humans [7] and *Anopheles funestus* Giles is one of the three major malaria vectors in Africa. It is the nominal member of a large group of mosquitoes that consists of at least eleven African species that are morphologically similar at the adult stage [8,9]: *An. funestus*, *An. funestus*-like, *An. vaneedeni*, *An. parensis*, *An. aruni*, *An. confusus*, *An. brucei*, *An. fuscivenosus*, *An. rivulorum*, *An. rivulorum*-like and *An. leesoni*[7-11]. *Anopheles funestus* is the most anthropophilic and endophilic member of the group [7], while the others are mainly zoophilic and not involved in malaria transmission except for *An. rivulorum,* which is a minor vector in Tanzania [12] and *An. vaneedeni* which is a possible vector under laboratory conditions [13].

*Anopheles funestus* and *An. rivulorum* are widely distributed throughout sub-Saharan Africa [7,9]. The extent of the distribution of the other members in the group is largely unknown or they are more localized. *Anopheles funestus*-like has so far only been recorded from Malawi [11] and *An. rivulorum*-like occurs in Cameroon and Burkina Faso but might extend to western and central Africa [9,14]. *Anopheles parensis* is found in eastern Africa, Swaziland and South Africa. *Anopheles confusus* is distributed in eastern and southern Africa and *An. vaneedeni* is found in South Africa. *Anopheles aruni*, *An. brucei* and *An. fuscivenosus* are extremely rare and found only in Zanzibar, Nigeria and Zimbabwe respectively [7,9].

A recent study of *An. funestus* population structure based on *NADH Dehydrogenase subunit 5* (*ND5*) mitochondrial DNA data [15] led to the description of two cryptic subdivisions, clade I which was found in all 11 African countries sampled and clade II found only in Mozambique and Madagascar. These two clades were differentiated by two fixed differences and an average of 2% divergence, which was thought to indicate that they have evolved independently for ~1 million years. Michel *et al.*[15,16] also reported the existence of at least one main division between populations of *An. funestus* on the basis of microsatellite allele frequencies. Furthermore, digestion of the internal transcribed spacer region 2 (*ITS2*) in the rDNA using restriction enzymes showed several different “types” within *An. funestus*[17,18] (see [19] for a review of the molecular systematics of *An. funestus*).

Understanding the characteristics of the species in the *An. funestus* group is necessary for effective malaria vector control programmes. The group may well be as complex and problematic as the *An. gambiae* complex [19,20] given the results that we have from only the few population genetic studies that have been done to date. The aim of the present study was to expand previous work by examining inter- and intra-specific relationships between the five most common members of the *An. funestus* group from southern Africa (*An. funestus*, *An. funestus*-like, *An. parensis*, *An. rivulorum* and *An. vaneedeni*), using mitochondrial markers and phylogenetic analysis.

## Methods

### Mosquito samples

Sampling information for the five species used in this study is given in Table 1. A total of 53 *An. funestus* specimens were collected resting inside houses from Mozambique (n = 32) in 2004 and Malawi (n = 21) in 2001. The collection of 26 *An. parensis*, 30 *An. vaneedeni* and 19 *An. rivulorum* specimens from South Africa were from outdoor CO_2_-baited traps between 2002 and 2008. The 27 *An. funestus*-like specimens from Malawi were collected resting inside houses in 2007 and F_1_ progenies from the specimens were used for this study. Different collection methods were used depending on the biology of each species to try and maximize species diversity.

**Table 1:** Species, localities and the total numbers of specimens for each species

	**Locality**	**Species**				
		***An. funestus***	***An. funestus*-like**	***An. parensis***	***An. rivulorum***	***An. vaneedeni***
Mozambique	Chibuto (24°40’S, 33°33’E)	32				
Malawi	Karonga (10°19’S, 34°08’E)	11	27			
	Nkhota kota (12°55’S, 34°18’E)	10				
South Africa	Mamfene (27°23’S, 32°12’E)			8		12
	Ndumu (27°02’S, 32°19’E)			18		
	Komatipoort (25°26’S, 31°57’E)				19	12
	Giyani area (23°15’S, 30°47’E)					6

### Laboratory methods

All specimens were identified to species by standard rDNA PCR methods [11,21,22]. DNA samples were extracted from either single mosquitoes or available parts of mosquitoes using standard extraction protocols [23].

The modified primers for *ND5* were from Michel *et al.*[16] and the primers for *COI* from Simon *et al.*[24]. The sequenced region for *ND5* was confirmed with the data from Michel *et al*. [16]. The region was amplified using primers New ND5F (5^′^-AGA AAT CAA TAT ATA GAA GAA GAT T-3^′^) and New ND5R (5^′^-TTC GAA TAT CTT GAG AAT TTT T-3^′^) for *ND5*, and C1-J-1718 (5^′^-GGA GGA TTT GGA AAT TGA TTA GTT CC-3^′^) and C1-N-2191 (5^′^-CCC GGT AAA ATT AAA ATA TAA ACT TC-3^′^) for *COI*. A total volume of 50 μL for each reaction contained 1 μL of the genomic DNA of an individual mosquito, 1X PCR Buffer, 2 mM MgCl_2_, 0.2 mM of each dNTP, 0.4 μM of each primer, and 1 unit of *Taq* DNA polymerase. PCR cycling conditions for *ND5* were as follows: a 5 minute 94°C denaturation step followed by 30 cycles of 45 seconds at 94°C, 45 seconds at 46°C and 1 minute at 72°C; there was a final extension step of 10 minutes at 72°C. Thermal cycling conditions for *COI* were initial denaturation at 94°C for 3 minutes, 35 cycles of denaturation at 94°C for 30 seconds, annealing at 48°C for 40 seconds and extension at 72°C for 30 seconds, and then a final extension at 72°C for 10 minutes.

A total of 155 DNA samples were sent to Macrogen Inc. in Korea and sequence analysis carried out using an ABI 3730XL DNA analyzer (Applied Biosystems, Foster City, CA).

### Data analysis

The DNA sequence data were aligned in Bioedit 7.0.9 [25]. The sequences were deposited in GenBank under accession numbers JQ424478-JQ424787. Sequence polymorphism and nucleotide divergence with Jukes and Cantor distance (K) statistics were estimated using DnaSP 5.0 [26]. Phylogenetic relationships for construction of a haplotype network were assessed using the statistical parsimony method implemented in TCS version 1.21 [27]. Neighbor-Joining (NJ) analysis was conducted using MEGA 4.0 [28]. Node support for NJ result was assessed using 1000 bootstrap pseudo-replicates. To find which substitution model best described the evolution of concatenated *ND5* and *COI* sequences, Modeltest [29] was used to perform a hierarchical likelihood ratio test. The Tamura Nei model [30] was specified for concatenated *ND5* and *COI* sequences using the Akaike information criterion. The model, TIM3 + I + G was used for maximum likelihood analysis using PhyML 3.0 [31]. The primer sequences were not added to the analysis due to unclear primer sequence data from some of specimens although the results were not affected.

## Results

Alignments of partial sequences from the 3′ end of the *ND5* (682 bp) and *COI* (524 bp) genes were analyzed from 155 individuals of *An. funestus*, *An. funestus*-like, *An. parensis*, *An. rivulorum* and *An. vaneedeni* identified using the Spillings *et al*. [11] and the Koekemoer *et al*. [21] methods (Table 1). All 21 *An. funestus* individuals from Malawi belonged to clade I while *An. funestus* from Mozambique showed the presence of both clades - 13 (40.6%) for clade I and 19 (59.4%) for clade II. The *An. rivulorum* results were distinct from the rest of the group and this species was used as an outgroup in subsequent analyses.

### Haplotype network analysis

**Figure 1:**
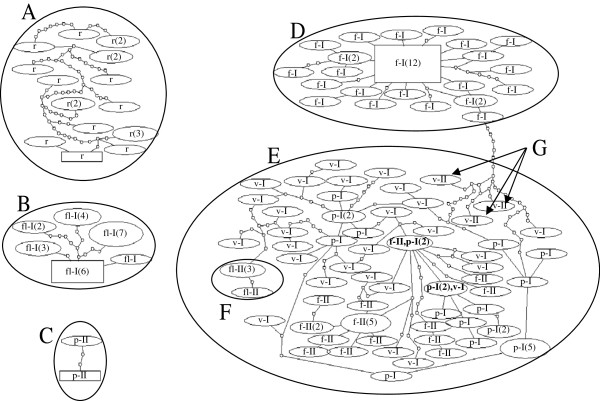
**Haplotype networks of mtDNA *ND5 *and *COI *concatenated sequences.** Haplotypes are represented as ovals, scaled to reflect frequencies. The most frequent haplotype (n = 1 in *An. rivulorum*, n = 6 in *An. funestus*-like, n = 1 in *An. parensis* excluded from the main *An. parensis* group and n = 12 in *An. funestus* subgroup), inferred as ancestral, is represented by a square. Lines connecting haplotypes and small ovals indicate one mutational step. **A**: *An. rivulorum*; **B**: *An. funestus*-like; **C**: *An. parensis* excluded from the main *An. parensis* group in clade II; **D***:* clade I in *An. funestus*; **E**: clade II in *An. funestus*, *An. parensis* clade I and *An. vaneedeni* clades I and II; **F**: *An. funestus*-like individuals excluded from the main *An. funestus*-like group; **G**: *An. vaneedeni* individuals excluded from the main *An. vaneedeni* group. f-I: *An. funestus* clade I; f-II: *An. funestus* clade II; fl-I: *An. funestus*-like clade I; fl-II: *An. funestus*-like clade II; p-I: *An. parensis* clade I; p-II: *An. parensis* clade II; r: *An. rivulorum*; v-I: *An. vaneedeni* clade I; v-II: *An. vaneedeni* clade II. Haplotypes marked in bold are shared between species. Figures in brackets are frequencies for each haplotype.

The concatenated sequence data of *ND5* and *COI* were analyzed using a statistical parsimony method [27] to construct an mtDNA haplotype network (Figure 1). Five main clusters emerged. The three clusters which contained *An. rivulorum* (Figure 1A), *An. funestus*-like (Figure 1B) and two *An. parensis* individuals excluded from the main *An. parensis* lineage (Figure 1C) were separated from the *An. funestus* subgroup lineages. The other clusters consisted of two lineages. One cluster included *An. funestus* clade I (Figure 1D). Mosquitoes included in clade I were within five mutational steps from the ancestral of clade I. The other cluster including *An. funestus* clade II (Figure 1E) was composed largely of haplotypes from *An. parensis* and *An. vaneedeni* as well as four *An. funestus*-like individuals (Figure 1F). There were two shared haplotypes: a) between *An. funestus* clade II and *An. parensis*; and b) between *An. parensis* and *An. vaneedeni* in Figure 1E. *Anopheles funestus* clade II samples were separated by multiple mutational steps from the clade I cluster. The cluster that contained the three *An. vaneedeni* haplotypes was located between the clade I and II clusters of *An. funestus* (Figure 1G).

### Neighbor-joining (NJ) and Maximum likelihood (ML) phylogenetic analysis

**Figure 2:**
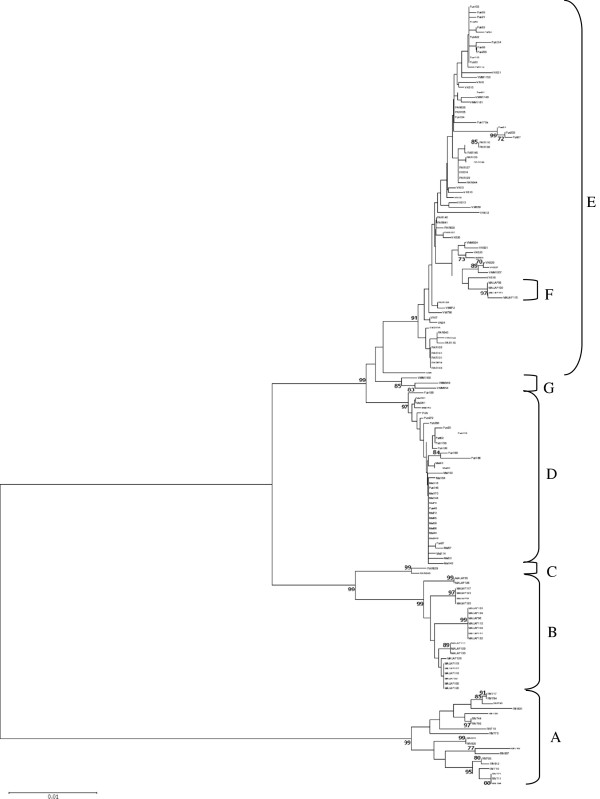
**Neighbor-joining tree.** The tree inferred from the concatenated *ND5* (a) and *COI* (b) loci with bootstrap percentages for 1,000 replicates and *An. rivulorum* as an outgroup. Bootstrap values under 70% are not shown. **A**: *An. rivulorum*; **B**: *An. funestus*-like clade I; **C**: *An. parensis* clade II; **D***: An. funestus* clade I; **E**: *An. funestus* clade II, *An. parensis* clade I and *An. vaneedeni* clades I; **F**: *An. funestus*-like clade II; **G**: *An. vaneedeni* clade II.

Sequence alignments of *An. funestus* (GenBank access No. DQ127052 for *ND5* and No. AY423059 for *COI*, specimens originating from Burkina Faso and Cameroon respectively) were used for the comparison in the phylogenetic trees. The concatenated sequence data of *ND5* and *COI* (total length 1206 bp) for the haplotype network and phylogenetic analysis are presented due to similar results with the individual data for both genes. The concatenated sequence data were analyzed using NJ analysis (Figure 2). Again the taxa were arranged in three distinct lineages: 1) *An. rivulorum*, 2) *An. funestus*-like clade I and *An. parensis* clade II, and 3) *An. funestus* clades I and II, *An. parensis* clade I, *An. vaneedeni* clades I and II, and *An. funestus*-like clade II. In *An. funestus* from Mozambique and Malawi, the results confirmed the two subdivisions, clade I and clade II. Four *An. funestus*-like and two *An. parensis* individuals that were excluded from the main species lineages (clade I) were designated *An. funestus*-like clade II and *An. parensis* clade II. Three *An. vaneedeni* specimens, which were separated from the main lineage (clade I), were designated *An. vaneedeni* clade II although they were included in the *An. funestus* subgroup lineage. The excluded samples were sequenced to confirm the species identification.

For the NJ tree of the *An. funestus* subgroup, *An. funestus* clade I was separated from the rest (*An. funestus* clade II, *An. parensis* clade I, *An. vaneedeni* clades I and II, and *An. funestus*-like clade II) with bootstrap values of 99%. *Anopheles parensis* clade II and *An. funestus*-like clade II were separated from *An. funestus*-like lineage (clade I) and *An. parensis* lineage (clade I) with bootstrap values of 99% and 97% respectively. Within the *An. funestus* subgroup, *An. vaneedeni* clade II was separated from the main *An. vaneedeni* lineage (clade I) with bootstrap values of 85%. The ML results are similar to the topology of the tree for the NJ results (Additional file 1).

### *ND5* sequence analysis

In the *An. funestus* group, *An. rivulorum*, which is the nominal member of the *An. rivulorum* subgroup, had the highest divergence from the other species while the nucleotide divergence with Jukes and Cantor distance (K) for *An. funestus*-like clade I was also high compared with the rest of the group (Table 2). *Anopheles parensis* clade II was separated from the *An. parensis* clade I (0.037) and close to *An. funestus*-like clade I (0.02). Table 2 shows a nucleotide divergence value of 0.023 between *An. funestus* clades I and II. The K values of *An. funestus* clade II against *An. funestus*-like clade II (0.008), *An. parensis* clade I (0.005), and *An. vaneedeni* clade I (0.006) were lower than the ones for *An. funestus* clade I against the same clades (Table 2). However, *An. vaneedeni* clade II had the same values (0.016) for the nucleotide divergence from both *An. funestus* clades. There were 9, 27 and 20 fixed different nucleotides between clades within *An. funestus*, *An. funestus*-like and *An. parensis* respectively (Table 3). Within the group, the highest polymorphic sites were found in *An. vaneedeni* clade I (34), and the highest number of fixed differences (27) between clades of *An. funestus*-like.

**Table 2:** **Nucleotide divergence (K) between species in the *An. funestus *group**

**Gene**	**Species**		***An. funestus***		***An. funestus-like***		***An. parensis***		***An. rivulorum***	***An. vaneedeni***	
			**I**	**II**	**I**	**II**	**I**	**II**		**I**	**II**
*ND5*	*An. funestus*	I	-								
		II	0.023	-							
	*An. funestus-like*	I	0.04	0.044	-						
		II	0.022	0.008	0.047	-					
	*An. parensis*	I	0.021	0.005	0.044	0.007	-				
		II	0.037	0.039	0.02	0.038	0.037	-			
	*An. rivulorum*		0.097	0.097	0.101	0.097	0.096	0.102	-		
	*An. vaneedeni*	I	0.021	0.006	0.044	0.008	0.005	0.038	0.097	-	
		II	0.016	0.016	0.039	0.018	0.013	0.034	0.097	0.014	-
*COI*	*An. funestus*	I	-								
		II	0.019	-							
	*An. funestus-like*	I	0.032	0.045	-						
		II	0.014	0.016	0.042	-					
	*An. parensis*	I	0.013	0.008	0.04	0.012	-				
		II	0.031	0.043	0.016	0.039	0.039	-			
	*An. rivulorum*		0.114	0.122	0.112	0.118	0.121	0.107	-		
	*An. vaneedeni*	I	0.014	0.009	0.04	0.011	0.007	0.037	0.12	-	
		II	0.008	0.022	0.038	0.017	0.017	0.033	0.12	0.017	-

Samples sizes are 44 (*An. funestus* clade I), 19 (*An. funestus* clade II), 23 (*An. funestus*-like clade I), 4 (*An. funestus*-like clade II), 24 (*An. parensis* clade I), 2 (*An. parensis* clade II), 19 (*An. rivulorum*), 27 (*An. vaneedeni* clade I) and 3 (*An. vaneedeni* clade II).

**Table 3:** **Summary of fixed differences and polymorphic sites between clades in the *An. funestus *subgroup**

		***An. funestus***		***An. funestus*-like**		***An. parensis***		***An. vaneedeni***	
		***ND5***	***COI***	***ND5***	***COI***	***ND5***	***COI***	***ND5***	***COI***
Polymorphic sites	Within Clade I	19	11	8	9	9	9	34	16
	Within Clade II	17	7	2	0	2	1	5	5
Fixed differences/ Polymorphic sites	Between clades I/II	9/37	6/21	27/37	18/27	20/29	15/23	0/36	3/22

### *COI* sequence analysis

The sequence analysis of *COI* between species in the *An. funestus* group and between each two clades within *An. funestus*, *An. funestus*-like, *An. parensis* and *An. vaneedeni* supported the *ND5* sequence analysis although the different nucleotides between clades within *An. vaneedeni* were 0 for *ND5* and 3 for *COI* (Tables 2 and 3). In the *COI* sequence analysis, the nucleotide divergence between *An. rivulorum* and the other species was slightly higher than the values from the *ND5* sequence analysis (Table 2). The nucleotide divergence values (K) were 0.019, 0.042, 0.039 and 0.017 between clades within *An. funestus*, *An. funestus*-like, *An. parensis* and *An. vaneedeni* respectively. Polymorphic sites for *COI* between clades were lower for *An. funestus* (21), *An. funestus*-like (27), *An. parensis* (23) and *An. vaneedeni* (22) than the ones for *ND5* (37, 37, 29 and 36 respectively) (Table 3). Comparison of our data with the *COI* data of Garros *et al.*[32] showed that the limited samples of Garros *et al.* (2–3 specimens) all fell within the main lineages described here, i.e. *An. funestus* clade I, *An. vaneedeni* clade I, *An. parensis* clade I and *An. rivulorum.*

## Discussion

The phylogenetic analysis of concatenated *ND5* and *COI* genes showed the existence of three distinct lineages for the five investigated species, i.e. 1) *An. rivulorum*, 2) *An. funestus*-like clade I and *An. parensis* clade II, and 3) *An. funestus* clades I and II, *An. funestus*-like clade II, *An. parensis* clade I and *An. vaneedeni* clades I and II. The results presented in this study support the hypothesis that there are at least two main divisions within *An. funestus* which is consistent with the results of Michel *et al.*[15] as well as the *An. funestus*-like study [11].

Previous studies of the *ITS2* and *D3* regions of the rDNA and *COI* and *Cytochrome Oxidase* subunit II (*COII*) mtDNA genes, for African and Asian *Anopheles* groups [32,33] showed that the *An. rivulorum* subgroup was clearly distinguished from members of the *An. funestus* subgroup (*An. funestus*, *An. parensis* and *An. vaneedeni*). Although there were slight differences in distances between members of the *An. funestus* subgroup reported in the two studies, the results reinforce the conclusion of distinct distance between *An. funestus* and *An. rivulorum* subgroups. Garros *et al.*[32] suggested that *An. rivulorum,* which is assigned to its own subgroup within the broader *An. funestus* group [34] and the *An. funestus* subgroup might have evolved from a common ancestor based on the phylogenetic trees of *ITS2*, *D3* and *COI*. It should be mentioned that *An. rivulorum* larvae are morphologically distinct from the members of the *An. funestus* subgroup [7] so it is not surprising that the molecular data should reflect this separation.

The phylogenetic data for *An. funestus*-like in this study suggest that it is a distinct lineage from the other species in the group. These results support Spillings *et al.*[11] showing this to be a new member of the *An. funestus* group. However, the phylogenetic trees from the concatenated *ND5* and *COI* both showed two clades for *An. funestus*-like. Four *An. funestus*-like specimens were separated into their own lineage with the rest falling into the *An. funestus* subgroup lineage. Furthermore, two *An. parensis* specimens were separated from the main *An. funestus* subgroup lineage and grouped with the *An. funestus*-like lineage. Further molecular investigations are needed to test the hypothesis of new species in the group and determine the relationship between *An. funestus*-like and the two *An. parensis* individuals.

In the study by Michel *et al.*[15], samples from East Africa had significantly lower average heterozygosity (0.455) and allelic richness (3.9) across all microsatellite loci, and lower mean mtDNA haplotype diversity (0.773) compared with the rest of Africa (0.606, 6.0 and 0.924 respectively). Other studies [16,35] have also reported two different subdivisions within *An. funestus* from the analysis of *ND5*. However, these proposed subdivisions were not correlated with the clades of Michel *et al.*[15]*.* Analysis of the mtDNA *cytochrome b* gene and the *ITS2* region in the rDNA [36] did not find any subdivisions within *An. funestus.* Previous studies using the RFLP method [17,18] that included samples of *An. funestus* from 16 African countries, found evidence for five genetic subdivisions on the *ITS2* and *D3* regions in the rDNA but again these were not correlated with the Michel *et al*. [15] clades.

In the phylogenetic trees in this study, the results did not show clear phylogeny between *An. funestus* clade II, *An. parensis* clade I and *An. vaneedeni* in the *An. funestus* subgroup even though these three were separated clearly from *An. funestus* clade I. This undistinguishing relationship between the three species may reflect a selective sweep or non-discrimination due to recent divergence that is known to occur in the mtDNA [37].

Surprisingly, *An. parensis* from South Africa shared two of the haplotypes, one with *An. funestus* from Mozambique and one with *An. vaneedeni* from South Africa. Donnelly *et al*. [38] reported that shared haplotypes between species in the *An. gambiae* complex might reflect non-contemporary processes such as incomplete lineage sorting between species or historical introgression events. So, although no natural hybridization between *An. funestus* clade II, *An. parensis* and *An. vaneedeni* has been reported, mitochondrial introgression may have happened through a recent event. However, Green and Hunt [39] reported that cross-mating experiments between *An. vaneedeni* and *An. funestus* resulted in sterile male hybrids and asynapsis of the giant polytene chromosomes, two phenomena that occur regularly in crosses between species of *Anopheles*[11,19,40]. A more likely explanation, therefore, is that these shared haplotypes are ancestral.

Although there were apparent subdivisions in the species studied here, these divergences may only be limited to mtDNA. The nature of mtDNA, i.e. haploid, maternal heritage and non-recombination, could retain both distinct mtDNA clades in admixed populations, while their nuclear genome would become homogenized. Michel *et al*. [15] reported that there was no corresponding nuclear divergence in spite of deep mtDNA divergence between clades I and II within *An. funestus*. They suggested that the subdivisions may result from historical introgression either among previously isolated and divergent populations or with a related species [15]. Additional research from other genomic regions is required to determine what these results mean in terms of specific status and relevance in epidemiology and to investigate their roles in malaria transmission in order to better understand the ecological aspects of this important vector group.

## Conclusions

Our findings indicate that five species in the *An. funestus* group comprises three lineages: A) *An. rivulorum*, B) *An. funestus*-like clade I and *An. parensis* clade II, and C) *An. funestus* clades I and II, *An. funestus*-like clade II, *An. parensis* clade I and *An. vaneedeni* clades I and II. This is the first step in the phylogenetic reconstruction of relationships between *An. funestus*-like and the other four common species of the *An. funestus* group, as well as between clades I and II of *An. funestus.* The study supports the conclusion that *An. funestus*-like is a new member of the *An. funestus* subgroup [11]. Intriguingly, the phylogenetic trees of the concatenated *ND5* and *COI* sequences showed that two subdivisions each for *An. funestus*-like, *An. parensis* and *An. vaneedeni* exist. Further investigations will be carried out to determine the specific status of the clades. In addition, further studies should also give insight into the roles played by the various species in malaria transmission.

## Competing interests

The authors declare no conflict of interest.

## Authors' contributions

KSC carried out the experiments and the analysis of the data and drafted the manuscript. MC and LLK designed the study, assisted with analysis of the data and helped draft the manuscript. All authors read and approved the final version of the manuscript.

## Supplementary Material

Additional file 1 **Maximum-likelihood tree.** The tree inferred from the concatenated *ND5* (a) and *COI* (b) loci with bootstrap percentages for 1,000 replicates and *An. rivulorum* as an outgroup. Bootstrap values under 70% are not shown. A: *An. rivulorum*; B: *An. funestus*-like clade I; C: *An. parensis* clade II; D*: An. funestus* clade I; E: *An. funestus* clade II, *An. parensis* clade I and *An. vaneedeni* clades I; F: *An. funestus*-like clade II; G: *An. vaneedeni* clade II. Click here for file
