# Supplementary material for: Population genetic structure of the major malaria vector Anopheles funestus s.s. and allied species in southern Africa
Source: Parasit Vectors. 2012 Dec 6;5:283. doi: 10.1186/1756-3305-5-283 (PMC3533957; doi:10.1186/1756-3305-5-283)
Supplement: Additional file 1 — Maximum-likelihood tree. The tree inferred from the concatenated ND5 (a) and COI (b) loci with bootstrap percentages for 1,000 replicates and An. rivulorum as an outgroup. Bootstrap values under 70% are not shown. A: An. rivulorum; B: An. funestus-like clade I; C: An. parensis clade II; D: An. funestus clade I; E: An. funestus clade II, An. parensis clade I and An. vaneedeni clades I; F: An. funestus-like clade II; G: An. vaneedeni clade II. [file 1756-3305-5-283-S1.pdf]

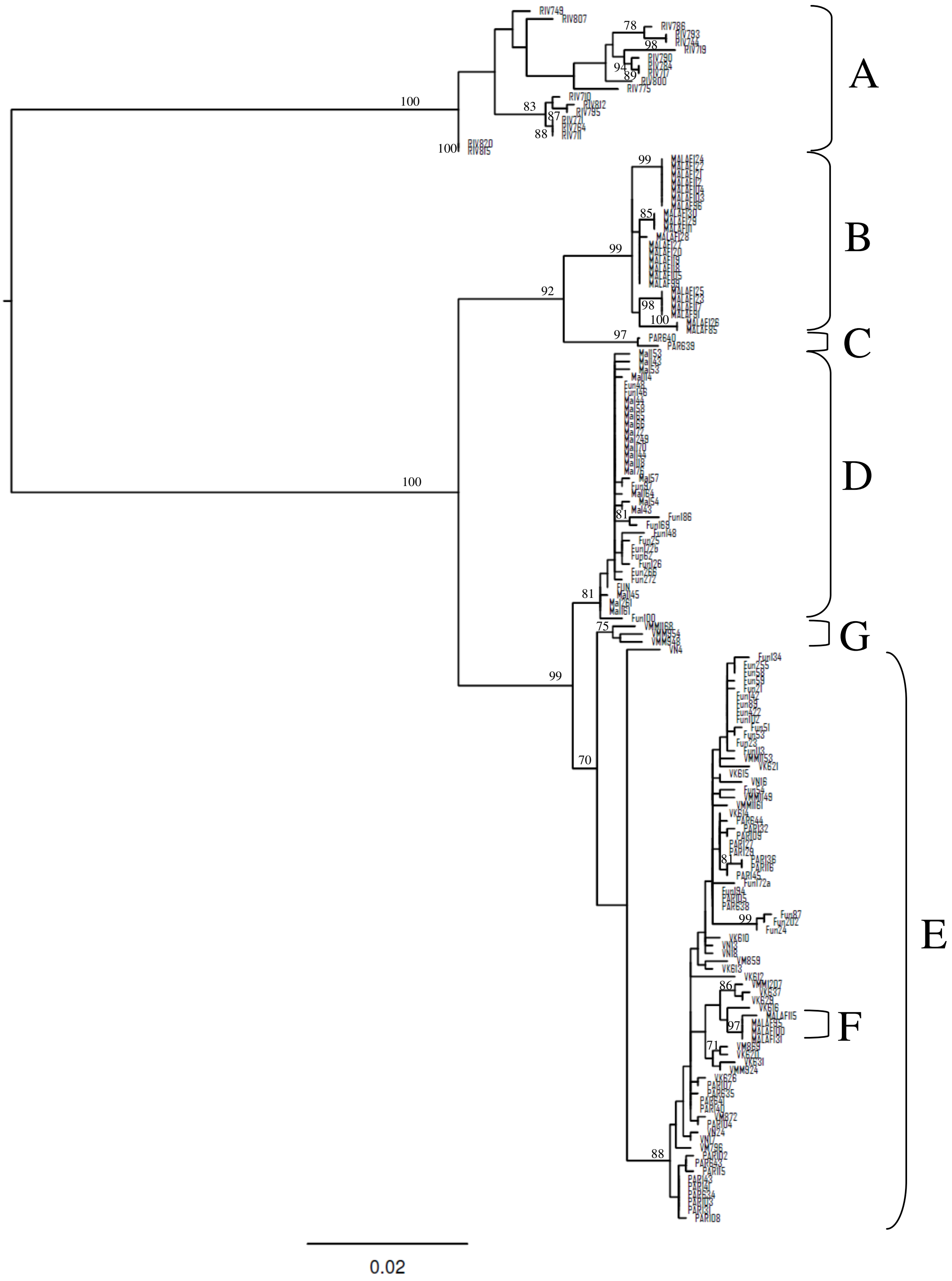
A
B
C
D
G
E
F
0.02

Supplementary 1. Maximum-likelihood trees.

The tree inferred from the concatenated *ND5* (a) and *COI* (b) loci with bootstrap percentages for 1,000 replicates and *An. rivulorum* as an outgroup. Bootstrap values under 70% are not shown. A: *An. rivulorum*; B: *An. funestus*-like clade I; C: *An. parensis* clade II; D*: An. funestus* clade I; E: *An. funestus* clade II, *An. parensis* clade I and *An. vaneedeni* clades I; F: *An. funestus*-like clade II; G: *An. vaneedeni* clade II.
